# Accelerating Structure–Property Relationship Discovery with Multimodal Machine Learning and Self-Driving Microscopy

**DOI:** 10.1021/acsnano.6c04715

**Published:** 2026-07-31

**Authors:** Jiawei Gong, Danqing Ma, Ralph Bulanadi, Robert Moore, Rama Vasudevan, Lianfeng Zhao, Yongtao Liu

**Affiliations:** † Center for Nanophase Materials Sciences, 6146 Oak Ridge National Laboratory, Oak Ridge, Tennessee 37830, United States; ‡ Department of Mechanical Engineering, Pennsylvania State University, Erie, Pennsylvania 16563, United States; § Holcombe Department of Electrical and Computer Engineering, 2545 Clemson University, Clemson, South Carolina 29634, United States; ∥ Materials Science and Technology Division, Oak Ridge National Laboratory, Oak Ridge, Tennessee 37830, United States

**Keywords:** self-driving laboratory, novelty discovery, representation learning, hybrid perovskites, atomic force microscopy

## Abstract

Microscopy combined with local spectroscopy is widely used to correlate nanoscale structure with functional properties in materials, but conventional measurements rely heavily on human-selected sampling locations and predefined targets, limiting data set diversity and the potential for discovery. Here, we present a framework that integrates autonomous microscopy with dual-novelty deep kernel learning (DN-DKL) for adaptive data acquisition and a dual variational autoencoder (VAE) for representation learning. DN-DKL actively guides the microscopy toward structurally and spectroscopically novel regions, enabling efficient collection of large spectral data sets. Dual-VAE embeds local structures and spectroscopic responses into a shared latent manifold that serves as a structure–property relationship map. We applied this framework for the investigation of halide perovskite films by using conductive atomic force microscopy. The results reveal distinct hysteresis behaviors that are linked to specific nanoscale structural motifs, including grain boundary junction points that show hysteresis under different bias conditions and asymmetric grain boundaries that suppress the charge transport. This framework establishes a general strategy that leverages the complementary strengths of self-driving microscopy, machine learning, and human expertise to accelerate scientific discovery in functional materials.

## Graphical abstract

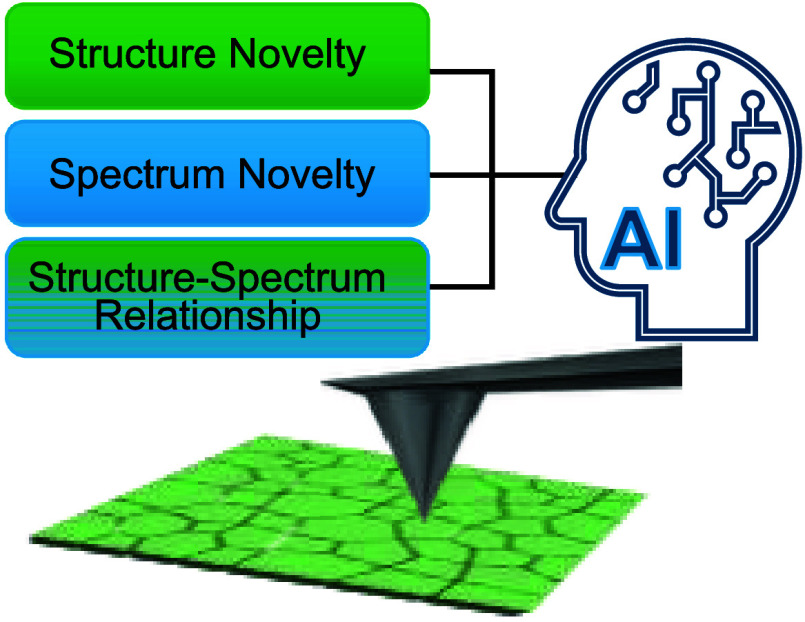

Microscopy has been a cornerstone in materials science and nanoscience, enabling researchers to investigate relationships between micro- and nanoscale structures and the resulting properties of functional materials.^1–4^ Microscopy image analysis combined with local spectroscopy is widely used to establish structure–property relationships in materials. For example, in ferroelectric materials, piezoresponse force microscopy imaging reveals the domain structure, while local piezoresponse spectroscopy analyzes polarization switching behavior such as nucleation voltage and coercive field at specific domains, directly linking domain structures to polarization switching dynamics.^5–7^ In nanoparticle samples, high-resolution scanning transmission electron microscopy images resolve atomic-scale defects, whereas spatially resolved electron energy-loss spectroscopy measures local band structure or carrier concentration, enabling the correlation between defect density and electronic transport properties.^8–11^ In two-dimensional (2D) materials,^12,13^ scanning probe microscopy (SPM) images lattice structure, edges, and wrinkles,^14^ while local conductive spectroscopy probes electronic density of states or excitonic behavior, directly connecting nanoscale structural features to local electronic properties.

Traditional correlative image and spectroscopy measurements in microscopy have relied on researchers’ expertise to guide where and what to measure, which are constrained by limited sampling, susceptibility to subjective bias, and the growing complexity of modern materials research. Recent advances in self-driving (or automated) microscopy offer a promising pathway toward more systematic exploration of structure–property relationships.^15–24^ Automated microscopes can operate continuously, performing vast experiments with consistency that exceeds human capabilities, generating extensive data sets that would be prohibitively time-consuming to acquire manually.^25,26^ Furthermore, machine learning (ML) algorithms, such as supervised ML and active learning,^27–33^ can be incorporated into automated microscopy workflows to adapt data acquisition in real time, enabling purpose-driven data collection. Supervised ML can recognize and classify predefined structural features within microscopy images, enabling targeted selection of regions for subsequent measurements.^34–37^ Supervised ML strategies are effective when the relevant structures and their significance are well understood, where it can guide the microscope toward structures deemed relevant based on prior knowledge and thereby streamlining data acquisition and improving efficiency. Active learning can guide experimental decisions by iteratively selecting new measurements that optimize a predefined property metric, such as maximizing conductivity, minimizing coercive field, or achieving a specific functional response.^38,39^ This approach is effective for directed optimization, but its reliance on predefined objectives inherently limits the scope of discovery. When focusing on predefined targets, active learning-based workflows may overlook unexpected phenomena or emergent behaviors.

In this work, we develop an integrated framework that combines the efficiency of automated microscopy for data acquisition, the capability of ML to disentangle complex, high-dimensional data sets, and the interpretive expertise of human scientists for structure–property relationship discovery. The framework incorporates a novelty-driven active-learning strategy to accelerate the acquisition of diverse and informative measurements. We then employ a dual variational autoencoder (VAE) approach to jointly analyze structural information from microscopy images and local spectroscopic responses for systematic exploration of structure–spectroscopy relationships. Finally, domain experts interpret the learned correlations to extract physical insights and guide the scientific understanding. We demonstrate the effectiveness of this approach by applying it to hybrid perovskite thin-film materials, where the relationship between grain structure and current–voltage (*IV*) behavior plays a critical role in optoelectronic performance. This integrated workflow resolves distinct nanoscale electrical regimes associated with grain interiors, grain-boundary grooves, and triple-junction nanotraps, revealing that hysteresis and charge transport are governed more strongly by localized geometric trap structures than by average grain size alone.

## Results/Discussion

### Multimodal MLs for Structure–Property Relationship Discovery

A key challenge in discovering structure–property relationships lies in the intrinsic complexity of how the material structure gives rise to functional behavior. This complexity arises because diverse structural features are often simultaneously coupled to multiple functional properties, making a comprehensive exploration difficult. Functional materials usually exhibit heterogeneity across multiple structural dimensions at once. For example, at any given location, a material may be characterized by a specific grain size,^40^ grain boundary character,^41^ crystal facet orientation,^42^ domain configuration,^43^ and defect density. These features vary spatially and interact with one another, producing a heterogeneous microstructural landscape in which each local region possesses a distinct structural identity and a local environment. In the meantime, a single material often exhibits multiple functional responses simultaneously, many of which can be encoded within the same local spectroscopic measurement and can be either dependent or independent. For instance, a polarization hysteresis loop can encode information about the polarization magnitude, coercive field, and nucleation voltage, while a current–voltage (*IV*) curve may reflect conductivity, turn-on voltage, saturation current, and hysteresis behavior. Each of these properties may be governed by different structural factors or complex interactions among multiple structural features. Consequently, structure–property relationships are rarely one-to-one; instead, they are inherently many-to-many (as shown in Figure 1) with intertwined dependencies between structural characteristics and functional responses.

**Figure 1:**
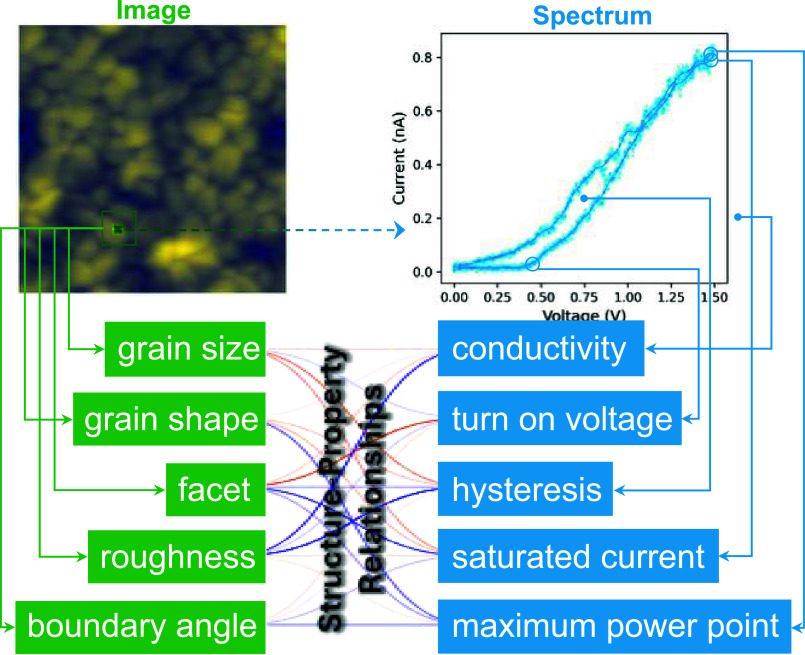
Complex, intertwined dependencies between structural characteristics and functional responses give rise to many-to-many structure–property relationships. Microscopy, combining high-resolution imaging with local spectroscopy measurement, is a powerful tool for probing these relationships at the micro- and nanoscale.

These considerations impose several key requirements on a framework aimed at discovering structure–property relationships. First, the framework must support the acquisition of structurally and functionally diverse data sets that adequately sample the heterogeneity of the material. Second, it must provide mechanisms to disentangle the complexity of the many-to-many structure–property mapping while minimizing information loss in both structural and property representations. To meet these requirements, we develop a framework (Figure 2a) that integrates automated microscopy with both a novelty discovery algorithm for accelerated data acquisition and representation learning using a variational autoencoder (VAE) for complex correlation analysis; the resulting outputs are then assessed and interpreted by human experts to derive physical insights.

**Figure 2:**
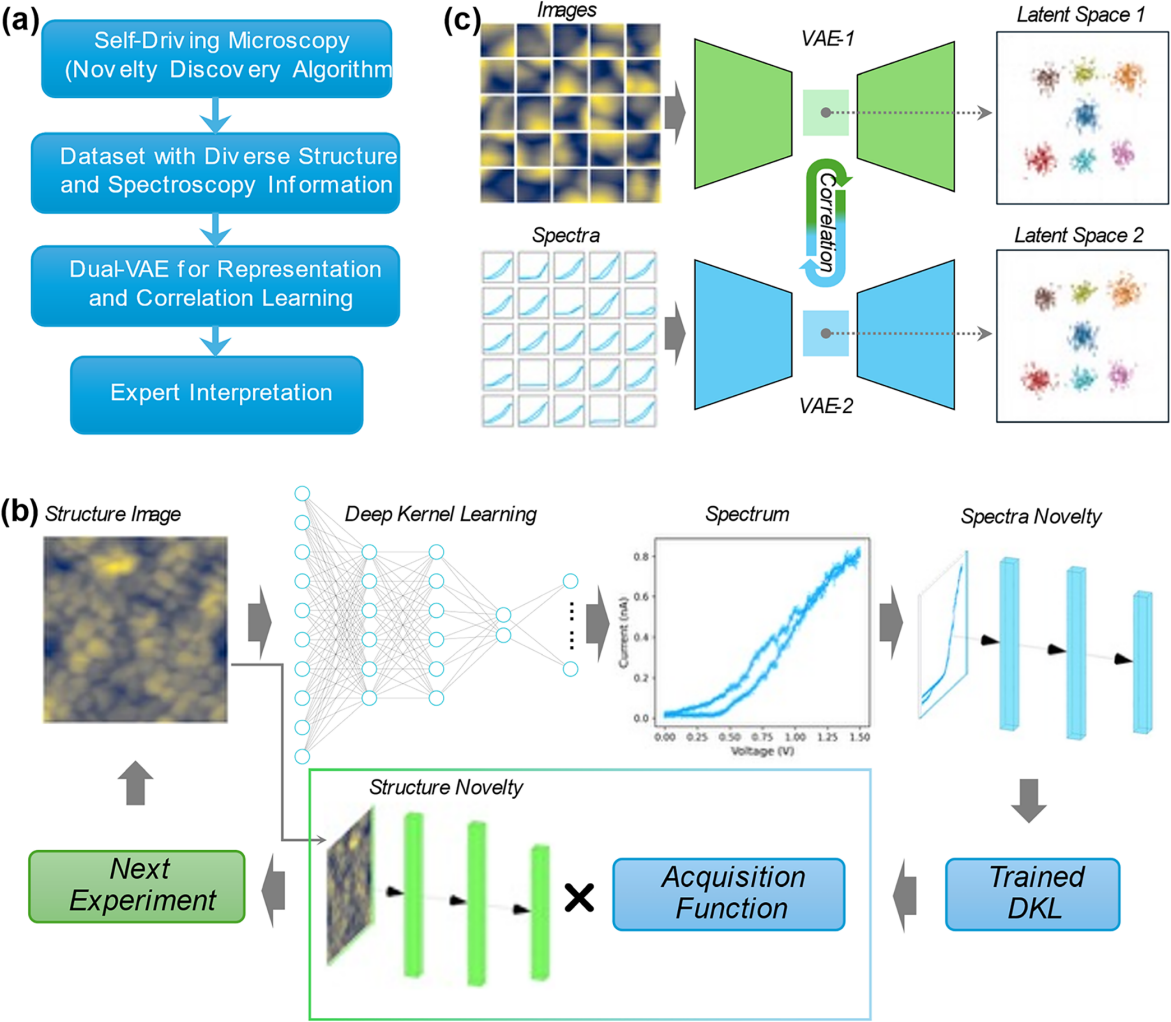
Dual-novelty deep kernel learning (DN-DKL) framework and Dual-VAE for structure–spectroscopy relationship investigation. (a) The workflow consists of DN-DKL for data acquisition and Dual-VAE for disentangling the structure–property relationship. (b) DN-DKL workflow integrating spectral and structural novelty scores to guide measurement selection. Spectral novelty prioritizes the acquisition of distinct spectra data, while structural novelty prioritizes measurements at structurally distinct locations. (c) Dual-VAE with parallel encoders for structural images and spectra, trained jointly to learn aligned latent representations and discover structure–property correlations.

We first develop an approach to acquire data sets that capture both structural diversity and rich spectroscopic information. To achieve this, we employ deep kernel learning (DKL),^38,44^ which has demonstrated strong capability in driving autonomous data acquisition in microscopy experiments.^45,46^ In prior implementations, DKL is typically guided by a predefined property descriptor, enabling the efficient optimization of the targeted property. While such a property-descriptor-driven approach allows exploration over a larger parameter space than manual measurements, it tends to follow narrow trajectories guided by predefined and specific metrics. As a result, it often fails to sample the broader landscape of structural and spectral information that extends beyond the predefined property metrics.

To overcome this limitation, we introduced novelty-based driving metrics into the DKL framework (Figure 2b). Specifically, we define a novelty score for spectroscopic measurements that quantifies how distinct a newly acquired spectrum is relative to previously collected data, thereby prioritizing the discovery of previously unexplored spectral responses.^47^ In addition, we also introduce a novelty score for structural images to refine the decision-making process in the DKL framework, prioritizing regions with structural features that are distinct from those already measured. The structure novelty score for each candidate location is defined as the complement of the maximum structural similarity index (SSIM) computed between its local image patch and all previously measured patches; therefore, a larger score indicates a patch that is structurally dissimilar to everything measured so far, while a smaller score indicates a patch that closely resembles already measured structures. The structure novelty scores for unmeasured structural images are incorporated into the DKL acquisition function via a gated active learning framework^48,49^ to jointly select the next measurement point. In this framework, the spectral novelty score is used directly as the training target for DKL, while the image-based novelty score is incorporated into the decision-making process to prioritize measurement at new structures. Together, this novelty-prioritizing strategy ensures the acquisition of both diverse spectroscopic data and diverse structural configurations, which are essential for establishing comprehensive structure–property relationships. More details about the spectrum and structure novelty scores, and the gated active learning framework can be found in Supporting Information Methods. Hereinafter, this approach will be termed dual-novelty-DKL (DN-DKL).

After acquiring structure images and spectra data, we employ a dual variational autoencoder (Dual-VAE)^50^ to learn correlations between structural images and spectroscopic data, as shown in Figure 2c. Dual-VAE consists of two parallel VAEs that encode structural images and spectra, respectively. Each VAE follows a standard encoder–decoder design, compressing high-dimensional inputs into low-dimensional latent distributions and reconstructing the original data. The two VAEs were jointly trained with aligned latent representations. By jointly optimizing reconstruction and latent consistency losses, the Dual-VAE learns a correlated structure–spectrum manifold that integrates complementary information from both structural images and spectroscopic data. This approach enables the disentangling of the structure–property relationships in complex material systems.

### Application to Halide Perovskite Thin Films

We applied this workflow to investigate halide perovskite thin films by using conductive atomic force microscopy (cAFM). Halide perovskites have promising applications in solar cells, light-emitting diodes, and photodetectors because of their exceptional optoelectronic properties. These materials exhibit complex nanoscale behavior, with spatially and temporally heterogeneous electrical responses. Local properties such as conductivity, band alignment, and recombination dynamics can vary significantly across a single film, despite uniform macroscopic measurements. This heterogeneity is closely associated with nanoscale and microscale features such as grains, grain boundaries, surfaces, and interfaces, where defect distribution, chemical composition, and lattice strain are often nonuniform. These regions can either facilitate charge transport or act as sites of ion accumulation and nonradiative recombination, and their roles may evolve dynamically under bias. cAFM is a powerful tool for investigating charge transport in perovskites at the nanoscale. In cAFM, topographic imaging captures the local structure of grains and grain boundaries, while localized *IV* measurements at selected positions reveal the electrical transport responses. By correlating *IV* characteristics with structural features, cAFM enables the investigation of the relationship between morphology and electrical response.

### Baseline Characterization of Perovskite Sample

We investigated a mixed-cation halide perovskite thin film, Cs_0.05_MA_0.05_FA_0.90_PbI_3_.^51^ Prior to the DN-DKL autonomous microscopy investigation, we performed baseline characterization of the perovskite sample to confirm material quality and establish relevant physical properties. Scanning electron microscopy (SEM) imaging (Figure 3a) reveals a compact, continuous morphology with dense grain packing, characteristic of high-quality polycrystalline films. Time-resolved photoluminescence (TRPL) measurement (Figure 3b) reveals the kinetics of carrier dynamics. By fitting the decay curve with a triexponential model,^52,53^ we found that the process is dominated by a fast component (τ_1_ = 1.28 ns; ∼59% amplitude), likely corresponding to rapid trap-assisted recombination at the interface or surface. This is accompanied by intermediate and slow components (τ_2_ = 6.81 ns and τ_3_ = 39.76 ns), yielding an average carrier lifetime (τ_average_) of 25.81 ns. The presence of the long-lived τ_3_ component indicates substantial bulk crystalline quality, allowing a significant portion of carriers to survive for collection. In addition, 2D PL lifetime mapping (Figure 3c) reveals spatial inhomogeneity in carrier lifetimes; these nanoscale variations underscore the necessity of detailed, spatially resolved structure–spectroscopy relationships investigated in this work. We note that a direct spatial correlation between the TRPL, 2D PL, and AFM-*IV* results would require correlative multimodal measurements that demand either dedicated instrumentation or substantial new experimental development, which could be a subject of future work. To further validate the quality of the hybrid perovskite film, we fabricated perovskite solar cells with a standard architecture of indium tin oxide (ITO)/MeO-2PACz/perovskite/C_60_/BCP/Ag (Figure 3d). The devices achieved a power conversion efficiency of 20.38%. Notably, this performance was obtained without additives or surface passivation layers, indicating that the measured characteristics primarily reflect the intrinsic behaviors of the perovskite material.

**Figure 3:**
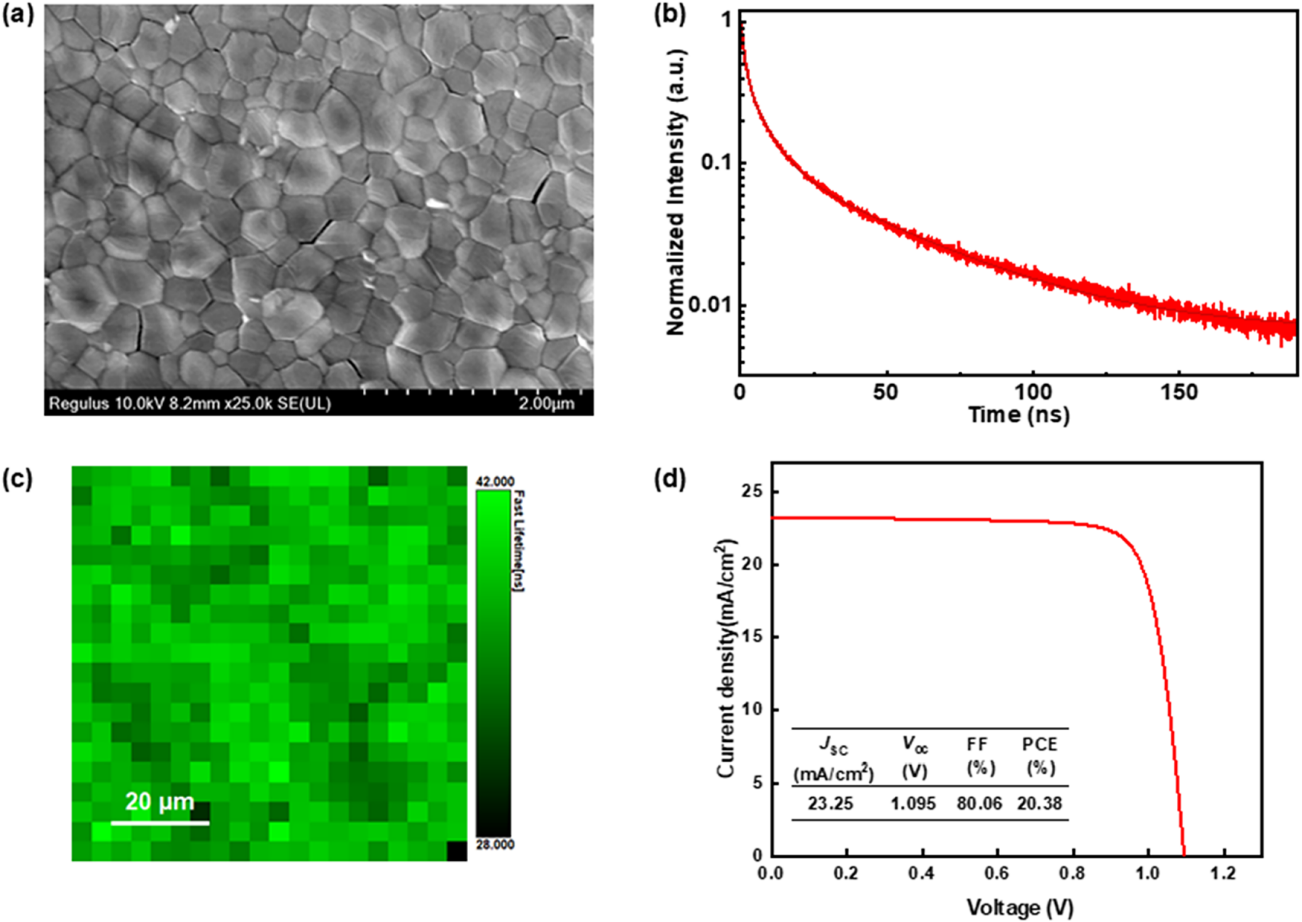
Characterization of the perovskite films and solar cell performance. (a) Top-view scanning electron microscopy (SEM) image of the perovskite film, displaying a dense and compact polycrystalline morphology. (b) Time-resolved photoluminescence (TRPL) decay kinetics of the perovskite film. (c) Spatially resolved PL lifetime mapping, highlighting nanoscale variations in carrier dynamics and film homogeneity. (d) Current density–voltage (*J*–*V*) curve of the perovskite solar cell measured under simulated AM 1.5G illumination.

### DN-DKL Autonomous cAFM Acquisition

We applied DN-DKL to cAFM-*IV* data acquisition on a halide perovskite thin film. Conventional cAFM-*IV* measurements rely on manual operation in which human operators position the AFM probe at selected locations to acquire *IV* data. This procedure inherently limits data set size and diversity, making a comprehensive investigation of structure–spectroscopy relationships impractical. In contrast, DN-DKL enables autonomous *IV* measurements that actively seek diverse and previously unseen behaviors. We implemented DN-DKL in our autonomous AFM system through our AEcroscopy platform,^15^ which enables seamless deployment of various ML approaches for automated and autonomous microscopy experiments. In the DKL setting, the model is trained using local topography image patches that capture micro- and nanoscale structural features and *IV* curves that encode the electrical response. The dual-novelty strategy ensures that the system prioritizes acquisition in regions that are simultaneously structurally unfamiliar and spectroscopically distinct from those of previously measured locations. As a result, the DN-DKL-driven autonomous cAFM samples rare features and underrepresented behaviors, producing a large, information-rich data set with broad morphological and electrical diversity for comprehensive analysis of structure–spectroscopy relationships.

Figure 4 shows the DN-DKL experimental process and results. Figure 4a shows the topography image containing structural features such as grains and grain boundaries. Overlaid on the topography are the *IV* measurement locations selected sequentially by DN-DKL. Starting from 10 seed points, 200 DN-DKL exploration steps were performed, yielding a total of 210 *IV* measurements. Figure 4b compares representative high-novelty and low-novelty *IV* curves against the mean *IV* curve; we plotted the top 5 high-novelty and 5 low-novelty *IV* curves. The high-novelty *IV* curves exhibit distinct features and deviate significantly from the mean *IV*; in contrast, the low-novelty *IV* curves closely resemble both the mean and each other. This suggests that spectral novelty-driven exploration effectively directs the measurement toward the discovery of *IV* characteristics distinct from previously sampled behavior.

**Figure 4:**
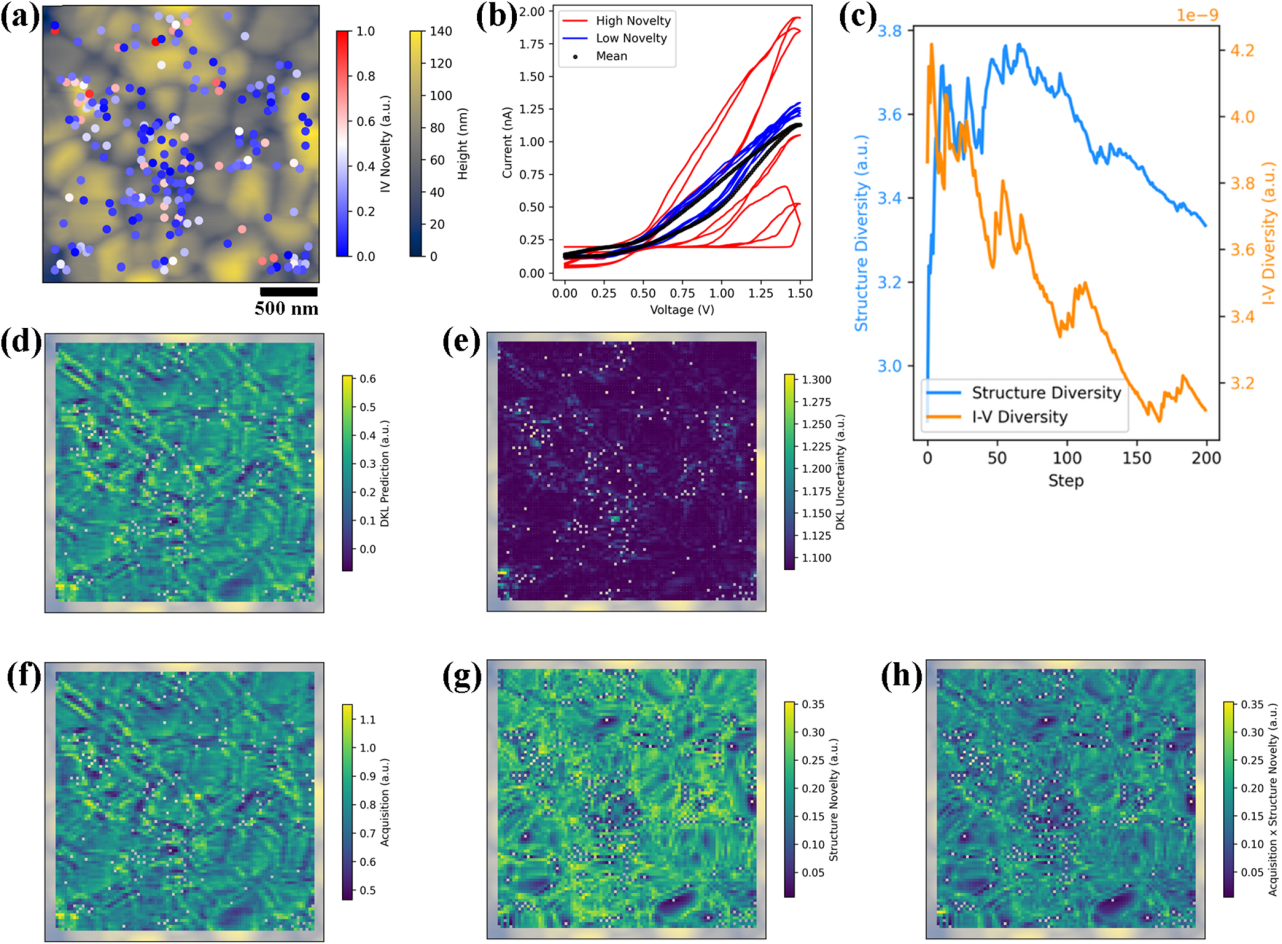
DN-DKL exploration of halide perovskite thin films. (a) Topography with overlaid *IV* measurement locations. (b) Representative high-novelty (top 5) and low-novelty (bottom 5) *IV* curves compared to the mean *IV* curve. (c) Structural and spectral diversity versus exploration step, showing an initial rapid increase followed by a gradual decrease with intermittent rises. (d, e) *IV* novelty prediction and uncertainty maps after 200 steps. (f–h) Acquisition function, image novelty score, and combined maps for unmeasured locations.

Data set diversity can be another metric for assessing the novelty-driven process. Figure 4c plots the diversity of both the structural patch set and the *IV* spectra set as a function of DN-DKL step, where diversity is quantified as the mean pairwise Euclidean distance across all samples in the data set (details about diversity calculation can be found in Supporting Information Methods). In our previous work,^47^ quantitative comparisons demonstrated that the novelty-driven active learning approach outperforms both random sampling and conventional physical-scalar-descriptor-driven DKL in terms of data set diversity, providing the benchmarking foundation upon which the present DN-DKL implementation is built. Here, both structural and spectral diversity increase drastically during the initial steps, reflecting effective novelty-driven exploration. After approximately 20 steps, diversity gradually decreases; notably, in a finite experimental space, overall data set diversity is expected to decrease after certain sampling steps. However, sporadic increases in both structural diversity and spectral diversity continue to occur, indicating that the novelty-driven approach continuously enhances the diversity of acquired data sets. The steps at which structural diversity and spectral diversity increase do not always coincide, suggesting that the structural novelty score and spectral novelty score contribute at different stages and work synergistically to maximize data set diversity.

Figure 4d,e shows the DN-DKL prediction and uncertainty maps after 200 experiments. The prediction (Figure 4d) is the spectrum novelty score predicted by the DN-DKL model at unmeasured locations, while the uncertainty (Figure 4e) reflects the DN-DKL’s confidence in its prediction. The predicted *IV* novelty shows a correlation with grain structure (Figure 4a), suggesting a strong structure–spectroscopy relationship that will be analyzed further later. Figure 4f–h shows the DN-DKL acquisition function, image novelty score map, and the combined map of acquisition and image novelty for unmeasured locations, respectively, which together illustrate the acquisition decision-making process. Figure 4f shows the acquisition values derived from the DKL prediction and uncertainty across all unmeasured locations. Notably, Figure 4f appears to be similar to Figure 4d, differing mainly in absolute scale. This similarity is because the model uncertainty (Figure 4e) is spatially uniform across most locations at this stage, and as such, the acquisition map is dominated by the model prediction, indicating that the model has converged to confident predictions and is operating predominantly in an exploitation regime. Figure 4g shows the structure novelty map, which clearly shows lower structural novelty around measured points (the white markers), indicating that the structural novelty effectively directs measurements toward new structural features rather than repetitively sampling similar structures. Figure 4h shows the adjusted acquisition values, obtained by multiplication of the acquisition values (Figure 4f) with the structure novelty scores (Figure 4g). The next measurement location is then selected as the position with the highest adjusted acquisition value in Figure 4h. This ensures that the final acquisition decision is jointly governed by both spectroscopic and structural novelty, prioritizing locations that score highly on both structure novelty and spectrum novelty. Together, DN-DKL effectively acquires data sets with diverse structural features and spectroscopic characteristics.

### Dual-VAE Structure–Property Relationship Analysis

Figure 5 shows the cAFM topography-*IV* data set of structural images and *IV* curves acquired by DN-DKL. Figure 5a is the topography overlaid with *IV* measurement locations, color-coded by sampling step. Figure 5b shows the structural patches from each measurement location. This image set reveals substantial structural diversity: some patches contain large grains, and some capture grain boundaries or boundary junction points. Grain shapes, flatness, and boundary widths also vary across the data set. Figure 5c shows the *IV* curves, which exhibit diverse characteristics including variations in current magnitude, current–voltage hysteresis behavior, and in some cases, negligible current response.

**Figure 5:**
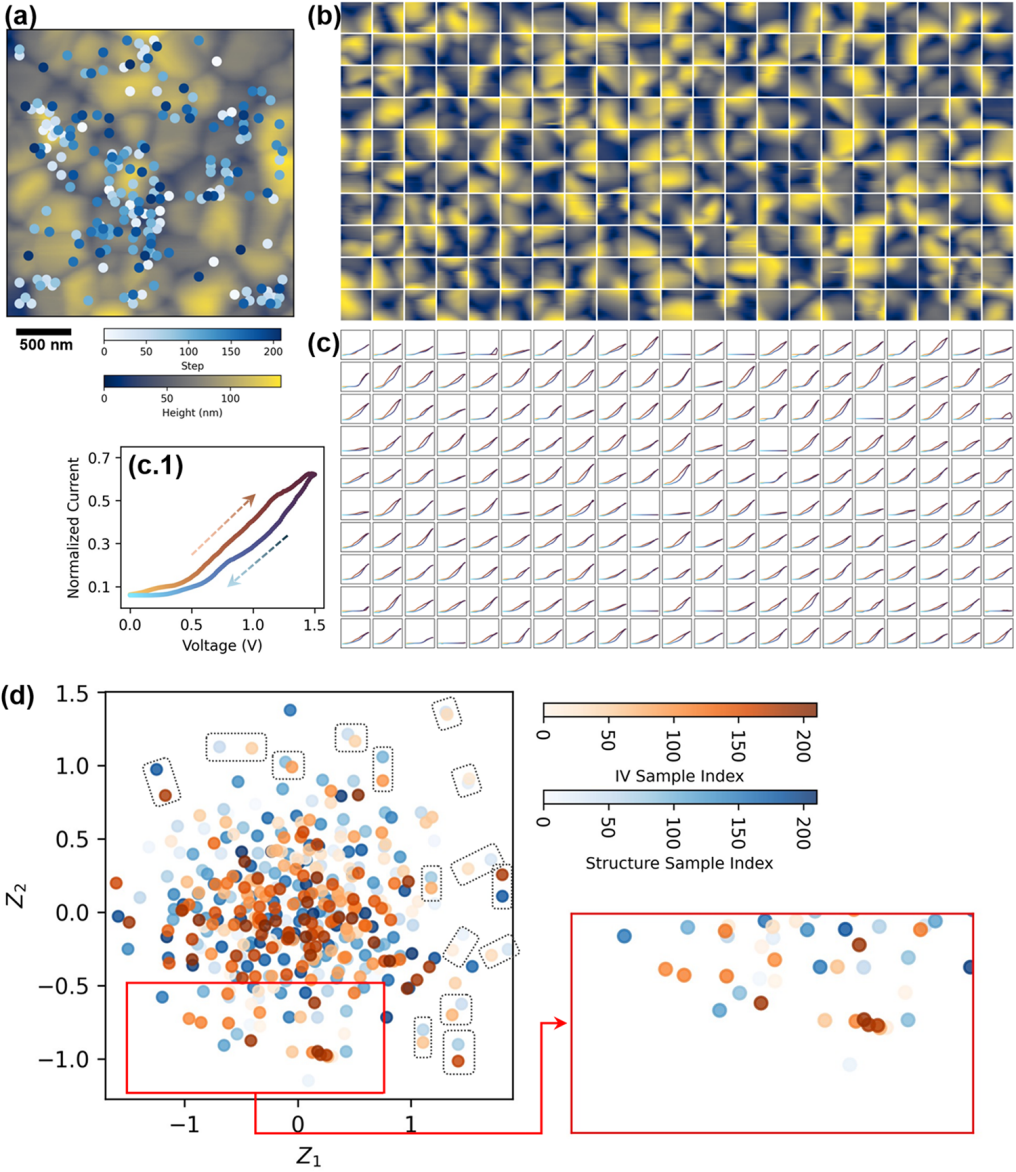
cAFM topography-*IV* data set. (a) Topography with color-coded measurement locations by sampling step. (b) Structural patches from the *IV* measurement locations showing diverse grain features and boundaries. (c) *IV* curves exhibiting varied current magnitudes, hysteresis, and conductivity (c.1) indicate the voltage sweep direction of *IV* curves. (d) Dual-VAE latent space indicates the learned correlation of structural features and *IV* responses, where a strong correlation between structure and *IV* is observed on top and right of the latent space (as exemplified by dashed boxes), but the bottom-left side indicates very weak correlation (as highlighted by the red box).

These results acquired by DN-DKL-driven autonomous cAFM capture both structural and electrical heterogeneity, enabling the investigation of the structure–property relationship using Dual-VAE. Figure 5d shows the combined latent space distribution, providing a joint representation of structural features and *IV* responses. Notably, the combined latent space is not just independent dimensionality reduction of structural features and *IV* responses; it also learns a coupled representation of the covariation between structural features and *IV* behaviors. Different regions of the latent space reveal different levels of structure-*IV* coupling. In the top and right regions, the image and *IV* samples with the same color value cluster closely together (as indicated by dashed boxes), indicating the Dual-VAE clearly captured their correlation. In contrast, the bottom-left region does not exhibit clear correspondence between structures and *IV* responses; here, similar structural features can lead to stochastic *IV* responses, or similar *IV* responses correspond to heterogeneous structures. After careful inspection, it indicates that in this region diverse structural features correspond to *IV* curves with very small measured currents, suggesting a many-to-one relationship. This behavior may originate from hidden variables not directly resolved in topography such as subsurface defects, ion accumulation, or compositional heterogeneity. Approximately 15–20 data points (∼10% of the total data set) in this region show weak structure–spectrum correlation. Because of the weak correlation, we would not draw a direct conclusion about the structure–property relationship from the bottom-left region in our further analysis. Nevertheless, the presence of this weak correlation can still be scientifically meaningful; it may point to missing or unobserved physical descriptors, or mechanisms governing *IV* behaviors that are not morphologically structure dependent. A more complete characterization including spatial mapping and cross-correlation with complementary measurements such as spatially resolved PL, AFM functional imaging, and chemical imaging would be a valuable direction for future investigation.

By embedding structural patches and corresponding *IV* curves into correlated manifolds, the model organizes the data set according to underlying physical correlation. Shown in Figure 6a,b are the image patches and *IV* curves generated by the trained Dual-VAE by traversing the latent space on a regular grid spanning [−1.64, 1.64] in both latent dimensions; this range is chosen to encompass the full distribution of the measured data points (Figure 5d). As a result, nearby points in the manifold correspond to regions that exhibit similar *IV* behavior (Figure 6b) associated with similar nanoscale structural features (Figure 6a). Examination of the correlated manifolds (Figure 6a,b) reveals both known physical behavior and previously underexplored phenomena.

**Figure 6:**
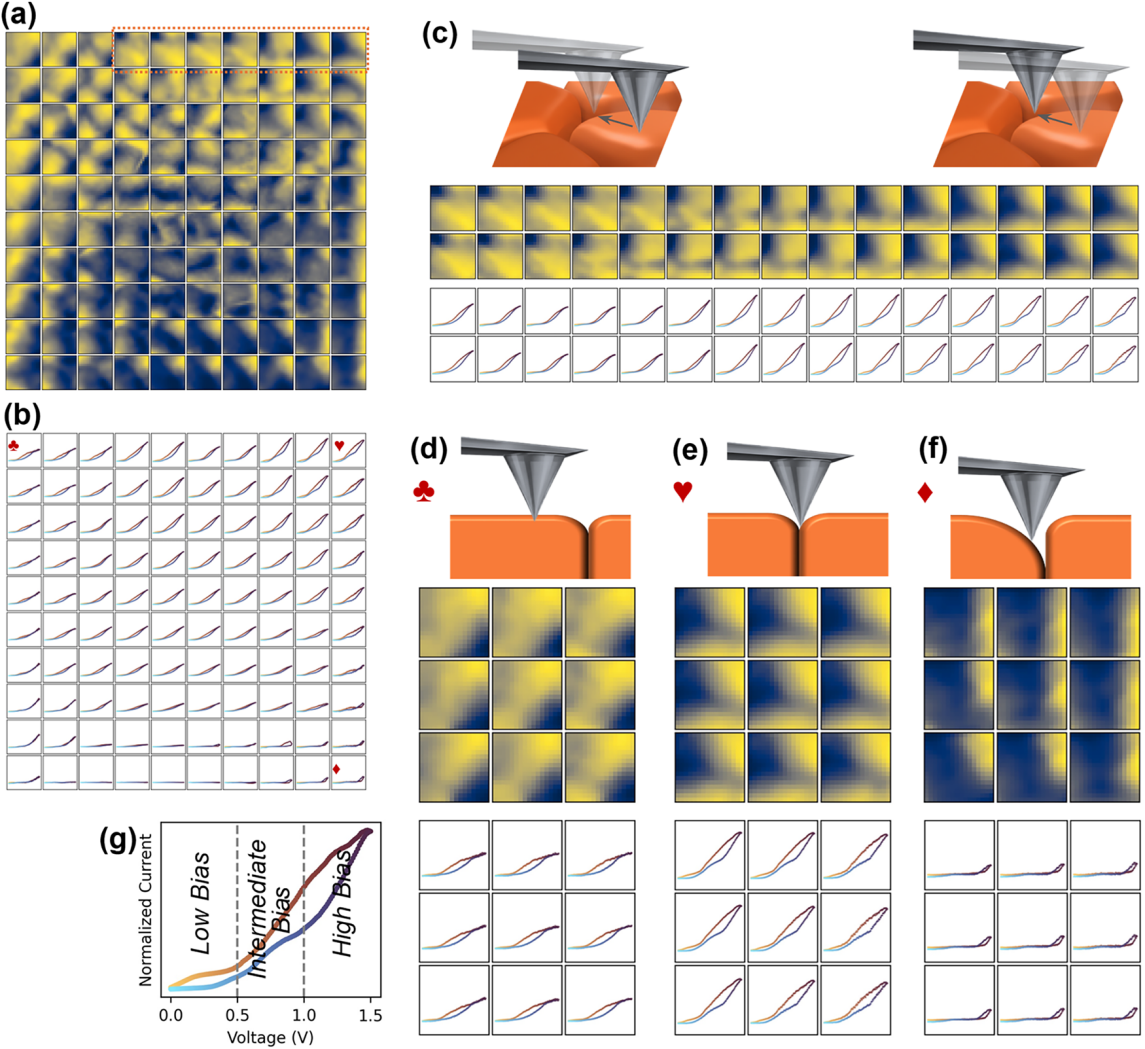
Correlated manifolds learned by the Dual-VAE linking structural features and *IV* curves. (a) Structural manifold, where each grid point represents a reconstructed structural feature corresponding to a latent coordinate. (b) *IV* manifold showing the reconstructed *IV* curves at the same latent coordinates as in panel (a), demonstrating a shared latent representation between structure and *IV* curves. The structural patches and *IV* curves in (a) and (b) are generated by the trained Dual-VAE by traversing the latent space on a regular grid spanning [−1.64, 1.64] in both latent dimensions. This range is chosen to encompass the full extent of the measured data distribution (Figure 5d). The patches are arranged left-to-right and bottom-to-top, corresponding to increasing values of the first and second latent dimensions, respectively. (c) Higher-resolution sampling corresponds to the region marked with the red box in panel (a) of the manifold illustrating a continuous transition of measurement locations from grain interiors to grain boundaries; the corresponding *IV* curves show progressively increasing hysteresis. (d–f) Representative *IV* hysteresis behaviors identified from panel (b) marked as “club,” “heart,” and “diamond” with their corresponding structural features. Symbols in panels (b) and (d–f) (club, heart, and diamond) indicate the correspondence between specific points in the overall latent space manifold (a, b) and the interpolated examples shown in panels (d)–(f). The club behavior is associated with grain interiors, the heart behavior with grain boundaries and triple junctions, and the diamond behavior with asymmetric grain boundaries that exhibit high turn-on voltage and suppressed current. Panel (g) indicates low, intermediate, and high bias conditions referred to in the discussion.

First, in the top right region (highlighted by a red box) of the structural manifold, a continuous transition from grain interiors to grain boundaries is observed. To better resolve this trend, we refined the sampling density of the manifold from a 10 × 10 grid to a 30 × 30 grid and extracted the corresponding structural patches and *IV* curves from matched spatial locations, as shown in Figure 6c. Notably, *IV* measurements were performed at the center of each structural patch. Moving from left to right in Figure 6c, the *IV* measurement locations (patch center points) gradually shift from grain interiors to grain boundaries. Correspondingly, the *IV* curves show a progressive increase in the current–voltage hysteresis. This trend suggests that grain boundaries play a significant role in modulating the electrical transport. The pronounced hysteresis is likely associated with trap states located in grain boundary grooves and/or junctions.^54^ This observed behavior is consistent with the established understanding that grain boundaries in halide perovskites contribute strongly to hysteretic transport,^55^ thereby supporting the physical validity of the learned manifold representation.

In addition to the continuous transition, the entire *IV* manifold (Figure 6b) reveals three distinct hysteresis behaviors, representative *IV* curves labeled as club, heart, and diamond. These are further illustrated in Figure 6d–f together with the corresponding structural features. Notably, the Dual-VAE enables dense interpolation across the full structures–spectra manifold. The interpolated results in Figure 6d–f reveal how local structural features and spectroscopic responses evolve continuously, providing a more complete picture of the structures–spectra correlation than discrete measured data points alone. The club behavior (Figure 6d) exhibits a hysteresis opening at intermediate bias (approximately 0.5–1.0 V); and the corresponding structure patches primarily indicate grain interiors. The heart behavior (Figure 6e) shows two hysteresis openings, one at low bias (∼0–0.5 V) and the other at intermediate-to-high bias (∼0.7–1.3 V); the corresponding structures are grain boundaries and triple-junction boundary points.^56^ We hypothesize that the double-opening hysteresis is due to the coexistence of two mechanisms: at lower bias, ion migration modifies the carrier extraction; at higher bias, progressive filling of trap states produces a second hysteresis at a distinct voltage. In contrast, the diamond behavior (Figure 6f) displays hysteresis at high bias (∼1.3–1.5 V); and the associated structures are asymmetric grain boundaries, where one side grain forms a sharp edge and the other side forms a smoother, deeper boundary, as indicated in the schematic illustration. At these asymmetric boundaries, the turn-on voltage of current is much higher (∼1.3 V) and the resulting current is strongly suppressed. The crystallographic mismatch at asymmetric grain boundaries can generate localized lattice strain and structural distortion, which lead to local changes in electron density and band structure. In addition, the resulting microstrain induces localized band bending, which promotes carrier trapping and the formation of deep trap states. Consequently, asymmetric grain boundaries act as active recombination centers that impede carrier extraction,^57^ leading to large turn-on voltage and reduced current in the *IV* curves. By comparison, the club and heart behaviors exhibit lower turn-on voltages (∼0.2 V) and higher resulting currents, indicating more efficient charge transport.

Overall, these results indicate that current–voltage hysteresis is strongly correlated to the presence and type of grain boundaries. Different boundary morphologies appear to produce distinct transport regimes, highlighting the importance of the nanoscale interfacial structure in governing local electrical behavior. The physical mechanisms associated with the variations of grain boundary shapes, such as defect accumulation or local band bending, require further attention in future investigations, but the learned manifold provides a systematic landscape for future study.

## Conclusions

In summary, we developed a framework for accelerating structure–property relationship discovery by integrating a discovery-driven workflow for autonomous data acquisition with correlative representation learning. The discovery-driven workflow, i.e., dual-novelty deep kernel learning (DN-DKL), actively selected measurement locations based on both structural and spectroscopic novelty to acquire data with unexplored phenomena, which enables efficient acquisition of information-rich data sets. In addition, a dual variational autoencoder (Dual-VAE) embedded local structural features and spectroscopic responses into a shared latent space, providing a correlative map that links nanoscale structures to functional behavior. We applied this framework to autonomous conductive atomic force microscopy measurements of halide perovskite thin films and revealed previously undersampled behaviors. The results revealed distinct current–voltage hysteresis responses associated with specific nanoscale motifs, including grain boundary junctions that exhibit bias-dependent hysteresis and asymmetric grain boundaries that suppress charge transport. These findings demonstrate how novelty-driven autonomous experiments combined with representation learning can accelerate scientific discovery. More broadly, this framework can be extended to incorporate other AI/ML-guided data acquisition strategies to enhance data set diversity and AI/ML-driven relationship discovery, hypothesis generation, and feature extraction. Future work could also extend the relationship analysis to multiple image channels and multiple spectra channels, allowing the ML model to identify an intertwined relationship between multiple images and spectra that is challenging for human experts to recognize. This strategy establishes a general pathway toward self-driving laboratories and faster discovery in complex materials by leveraging the efficiency of autonomous data acquisition, ML analysis, and human interpretation, which can be applied to a wide range of material systems and multimodal characterization techniques.

## Methods/Experimental

### Conductive AFM

Conductive atomic force microscopy (cAFM) measurements were performed in an Asylum Research Cypher AFM instrument (Oxford Instruments) using a Pt/Ir-coated AFM tip (ElectriMulti75-G (Budget Sensors)) and a dual-gain ORCA tip holder. DN-DKL was implemented in the Cypher AFM via the AEcroscopy platform (https://yongtaoliu.github.io/aecroscopy.pyae/welcome_intro.html) that was developed for AI/ML-driven autonomous microscopy experiments.

### Perovskite Film Preparation

The perovskite film was prepared from a precursor solution of formamidinium iodide (FAI), methylammonium iodide (MAI), cesium iodide (CsI), and PbI_2_ dissolved in a DMF/DMSO mixed solvent (4:1 v/v). The films were deposited onto ITO substrates modified with a [2-(3,6-dimethoxy-9*H*-carbazol-9-yl)­ethyl]­phosphonic acid (MeO-2PACz) self-assembled monolayer, serving as an efficient hole transport layer.

## Supplementary Material



## Data Availability

Data and code for this work are available in the manuscript, Supporting Information, or at Github: https://github.com/yongtaoliu/DNDKL_DVAE_PVSK.

## Notes

Experimental methods including perovskite preparation, device fabrication, solar cell characterization, scanning electron microscopy, time-resolved photoluminescence, conductive AFM, spectrum novelty, structure novelty, diversity metric, Dual-VAE, and image patches preparation (PDF)
