# Supplementary material for: Accelerating Structure–Property Relationship Discovery with Multimodal Machine Learning and Self-Driving Microscopy
Source: ACS Nano. 2026 Jul 31;20(31):21827–38. doi: 10.1021/acsnano.6c04715 (PMC13472328; doi:10.1021/acsnano.6c04715)
Supplement: Supplementary file 1 [file nn6c04715_si_001.pdf]

Supplementary Information

Accelerating Structure-Property Relationship Discovery with Multimodal Machine Learning and Self-Driving Microscopy

Jiawei Gong[1,2], Danqing Ma[3], Ralph Bulanadi[1], Robert Moore[4], Rama Vasudevan[1], Lianfeng Zhao[3], Yongtao Liu[1]*

[1] Center for Nanophase Materials Sciences, Oak Ridge National Laboratory, Oak Ridge, TN, 37830, USA

[2] Department of Mechanical Engineering, Pennsylvania State University, Erie, PA, 16563, USA

[3] Holcombe Department of Electrical and Computer Engineering, Clemson University, Clemson, SC, 29634, USA

[4] Materials Science and Technology Division, Oak Ridge National Laboratory, Oak Ridge, TN, 37830, USA

* Corresponding Author Email: liuy3@ornl.gov

Methods

Perovskite Precursor Preparation

Formamidinium iodide (FAI) and methylammonium iodide (MAI) were purchased from Greatcell Materials. Lead(II) iodide (PbI_2, 99.99%) was purchased from TCI. Cesium iodide (CsI), N,N-dimethylformamide (DMF), dimethyl sulfoxide (DMSO), isopropanol (IPA), and chlorobenzene (CB) were obtained from Sigma-Aldrich. The self-assembled monolayer (SAM) material, [2-(4-methoxyphenyl)ethyl]phosphonic acid (MeO-2PACz), was purchased from Luminescence Technology Corp. (Lumtec). Fullerene (C60) and Bathocuproine (BCP) were purchased from Xian Polymer Light Technology Corp. All chemicals were used as received without further purification. The perovskite precursor solution (1.5 M) was prepared by dissolving 691.5 mg of PbI_2, 232.16 mg of FAI, 11.92 mg of MAI, and 19.49 mg of CsI in 1 mL of a mixed solvent of DMF and DMSO (4:1 volume ratio). The solution was stirred at room temperature until fully dissolved and filtered through a 0.45 μm PTFE filter prior to use.

Device Fabrication

Hole Transport Layer (HTL): The MeO-2PACz SAM solution was prepared by dissolving the powder in IPA at a concentration of 0.3 mg/mL. The SAM solution was spin-coated onto the substrate at 3000 rpm for 30 s with an acceleration of 1500 rpm/s.

Perovskite Active Layer: The perovskite films were deposited using a two-step spin-coating program: (1) 2000 rpm for 10 s (acceleration 200 rpm/s) and (2) 4000 rpm for 40 s (acceleration 1500 rpm/s). During the second step, 150 μL of CB antisolvent was dripped onto the center of the substrate at the 35th second from the start of the program. The films were then immediately annealed on a hotplate at 100 ℃ for 30 min.

Electron Transport Layer (ETL) and Electrode: The devices were completed by thermally evaporating C60 (30 nm), BCP (8.5 nm), and Ag (85 nm) in a thermal evaporator. The deposition rates were controlled at 0.3 Å/s for both C60 and BCP, and the Ag electrode was evaporated at a rate of 0.6 Å/s.

Solar Cell Characterization

Photovoltaic performance was evaluated by measuring current density-voltage (*J*-*V*) curves under simulated AM 1.5G illumination (100 mW/cm^2). A MiniSol Sun Simulator (Model LSH-7320, Newport Corporation) was used as the light source, with the intensity calibrated using a standard silicon reference cell (Model 91150V, Newport Corporation). Data acquisition and voltage sweeps were performed using a Keithley 2400 SourceMeter Unit (SMU).

Scanning Electron Microscopy

Surface morphology was characterized using a Hitachi SU 8230 ultra-high resolution cold-field emission scanning electron microscope (FE-SEM).

Time-Resolved Photoluminescence

Spatially resolved PL lifetime mapping and decay kinetics were measured using a time-resolved fluorescence microscope (MicroTime 100, PicoQuant). The PL decay curves were fitted using a standard tri-exponential decay model to extract the carrier lifetimes:

$$I(t) = A_1\, exp\left(-\frac{t}{\tau_1}\right) + A_2\, exp\left(-\frac{t}{\tau_2}\right) + A_3\, exp\left(-\frac{t}{\tau_3}\right)$$

where $I(t)$ is the PL intensity at time t, A_i represents the fractional amplitude of each decay component, and τ_i denotes the corresponding carrier lifetime. The average carrier lifetime ($\tau_{average}$) was calculated using the amplitude-weighted equation: $\tau_{average} = \sum A_i\tau_i^2/\sum A_i\tau_i$ [47].

Spectrum Novelty

The spectrum novelty score quantifies how anomalous a newly measured IV curve is relative to previously acquired spectra. At each iteration, all normalized IV curves are passed to an Isolation Forest (IF) model from scikit-learn. The IF assigns an anomaly score to each spectrum based on how few partitions are required to isolate it from the rest of the dataset, spectra that are distinct receive higher novelty scores. Notably, the IF is refit at every iteration as new spectrum is added, so the definition of novelty evolves dynamically as the dataset grows. The resulting scores are normalized to [0, 1] and used as the target values for the DKL surrogate model.

Structure Novelty

The structure novelty score quantifies how structurally distinct an image patch (i.e., an unmeasured location) is from all locations that have already been measured. Let $R = \{r_1, \ldots, r_N\}$ denote the set of measured image patches and t_i denote a candidate image patch from the unmeasured patch set. For each candidate patch, the Structural Similarity Index (SSIM) is computed with respect to every measured patch,

$$S_{ij} = SSIM(t_i, r_j)$$

and the maximum similarity value is retained,

$$s_i^{max} = \max_{r_j \in R} S_{ij}$$

The structure novelty score is then defined as

$$N(t_i) = 1 - s_i^{max}$$

where $N(t_i)$ denotes the novelty of candidate patch t_i. A novelty score close to 1 indicates that the candidate patch is structurally distinct from all previously measured patches, suggesting that it may provide new information if selected for measurement. In contrast, a score close to 0 indicates strong structural similarity to at least one previously measured patch and therefore limited expected information gain.

To maintain computational efficiency during live experimentation, the full pairwise SSIM matrix between the target and reference sets is computed once during initialization. After each acquisition step, when a target patch is promoted to the measured set, the SSIM matrix is updated incrementally by computing only the similarities between the newly measured patch and the remaining unmeasured patches. This procedure appends a single column to the cached SSIM matrix, reducing the per-iteration computational cost from a full matrix recomputation to a linear-time update with respect to the number of remaining candidate patches.

Gated Active Learning Framework

The gated-active-learning (GAL) framework is employed to balance spectrally informative regions and structurally diverse regions. The GAL consists of two coupled loops. The primary Bayesian

optimization (BO) loop evaluates spectrum novelty and uncertainty to identify candidate measurement locations, while a secondary loop assesses the structural diversity of the image space. Structural diversity is quantified using a structural novelty score as illustrated above. The structural novelty information is incorporated at the acquisition stage through a gating mechanism. Rather than selecting measurement locations solely according to the BO acquisition function, the acquisition values are adjusted using the structural novelty score. Let $A_{BO}(t_i)$ denote the acquisition value generated by the primary BO model from the predicted spectrum novelty and uncertainty. The structure novelty score is incorporated as a gating factor that modulates the acquisition value according to $A_{gated}(t_i) = A_{BO}(t_i)N(t_i)$. This gating strategy promotes simultaneous exploration of spectral and structural diversity.

Diversity Metric

Dataset diversity is computed as the mean pairwise Euclidean distance across all samples in the dataset, calculated using pairwise_distances from sklearn.metrics with metric = 'euclidean', which returns a symmetric n × n distance matrix where each entry represents the Euclidean distance between two samples in feature space, the mean is taken over all entries of the matrix to obtain a diversity score; a higher diversity score indicates that the collected dataset spans a broader and more representative range.

Dual-VAE

The Dual-VAE framework employs two parallel variational autoencoders (VAEs), one for image patches and the other for spectra, each consisting of a encoder and decoder. The image VAE encoder outputs 5 dimensions total (2 for latent content + 3 for coordinate transformations: 1 rotation angle and 2 translational parameters), while the spectral VAE encoder produces a 2-dimensional latent vector directly. Dual-VAE is trained jointly with a loss function $\mathcal{L} = -(\mathcal{L}_{reconstruction} - \beta \cdot KL) + \gamma \cdot \mathcal{L}_{l1}$; where β = 1 and γ = 10 (weight on the latent alignment loss), binary cross-entropy was used as the reconstruction loss for both image patches and spectra.

Image Patches Preparation

The full AFM image was divided into patches using a sliding window approach, where a fixed-size window was swept across the image, with each window position centered on a pixel location to yield one image patch. Helper functions for generating image patches are available in github https://github.com/yongtaoliu/DNDKL_DVAE_PVSK.
